# Tuberculosis of acromioclavicular joint: a case report

**DOI:** 10.1186/s12879-019-3760-6

**Published:** 2019-02-04

**Authors:** Jian Cheng, Shiming Feng, Huining Lei, Weiling Huo, Huanhuan Feng

**Affiliations:** 1 Department of Orthopedics, The Affiliated Xuzhou Hospital of Medical College of Southeast University (Xuzhou Central Hospital), No.199, Jiefang South road, Quanshan District, Xuzhou, 221009 Jiangsu Province China; 2 The First Clinical Medical College, Nanjing University of Chinese Medicine, No.138, Xianlin road, Qixia District, Nanjing, 210046 Jiangsu Province China; 3 Department of Orthopedics, The Affiliated Taicang Hospital of Nanjing University of Chinese Medicine, No.140, People’s South road, Taicang, Suzhou, 215400 Jiangsu Province China

**Keywords:** Tuberculosis, Osteoarticular tuberculosis, Acromioclavicular joint, Arthritis

## Abstract

**Background:**

Osteoarticular tuberculosis is a great masquerader presenting in varied forms and in atypical locations, and it is prone to misdiagnosis and missed diagnosis. Isolated acromioclavicular joint tuberculosis has been reported rarely.

**Case presentation:**

A 19-year-old man presented with a chronic, mild pain, non-healing ulcer in right shoulder. Imaging of the shoulder revealed destruction of the acromioclavicular joint and histopathology confirmed the diagnosis of acromioclavicular tuberculosis. The patient underwent debridement, synovectomy and drainage of the abscess and recovered well with antitubercular therapy postoperatively.

**Conclusions:**

Awareness of this uncommon presentation of osteoarticular tuberculosis may assist in earlier diagnosis. Especially, in endemic countries, osteoarticular tuberculosis should be considered as a differential diagnosis in all atypical presentations to avoid residual problems.

## Background

Tuberculosis is a refractory disorder that can easily lead to loss of workforce. The osteoarticular tuberculosis accounts for a small percentage of tuberculosis. It presents diagnosis and management challenges for its usually combined with atypical presentations, especially when involves the non-weight bearing joints. There have been less than 10 relevant case reports about acromioclavicular joint tuberculosis, and it is prone to misdiagnosis and missed diagnosis. We present our rare osteoarticular tuberculosis case in the acromioclavicular joint.

## Case report

A 19-year-old man presented to our outpatient department with mild pain and swelling at the right shoulder for 3 weeks. There was no preceding history of trauma. The pain, mild in intensity, increased during shoulder movement. There was no history of fevers, night sweats and weight loss. And no previous history of tuberculosis or contact with persons suffering tuberculosis was recorded. The lesion started as a papule and ulcerated with copious purulent discharge for 10 days when the patient presented to our department. Dressing change and anti-infective drug (cephalosporin) were proved ineffective in other hospitals.

On physical examination, there was mild tenderness present over the anterior aspect of the right shoulder. A sinus was seen at the distal of the clavicle, sized 1.5 cm × 2.0 cm, 2.0 cm in-depth, with a purulent base, surrounding erythema and induration. There was a lot of canary yellow purulent discharge in the sinus. Full active range of motion was observed at the shoulder. There were no palpable lymph nodes and systemic examination did not reveal any abnormalities.

**Fig. 1:**
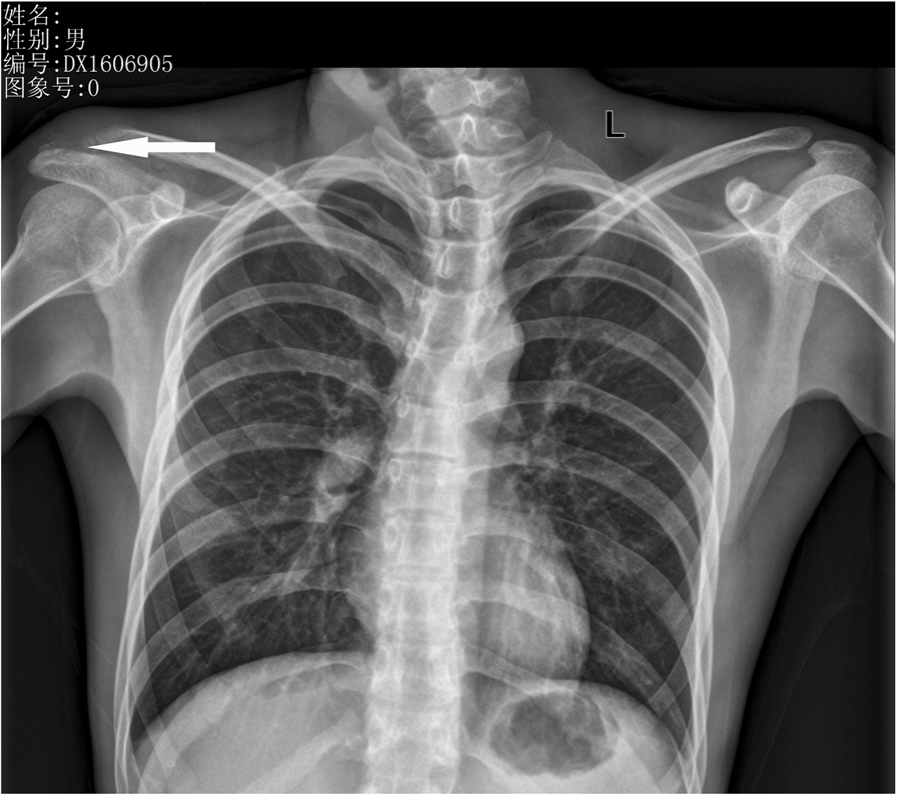
X-ray showing erosions at the acromion process of scapula

**Fig. 2:**
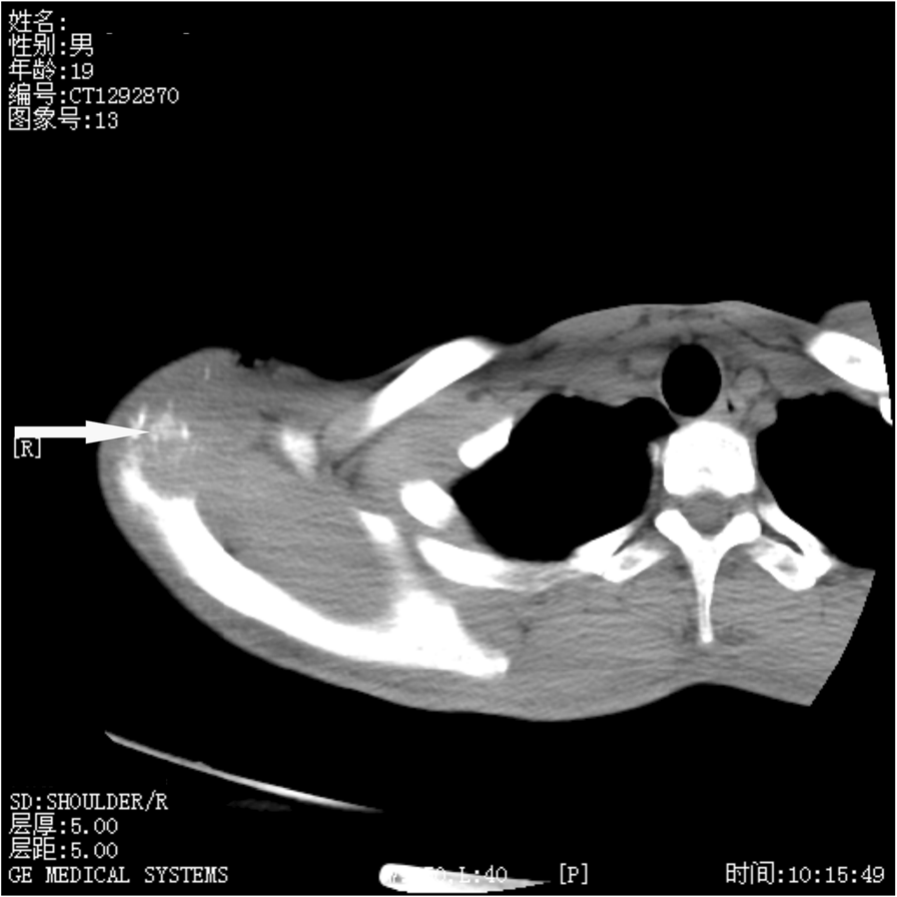
CT scan of right acromioclavicular joint showing erosions at the acromion process of scapula

**Fig. 3:**
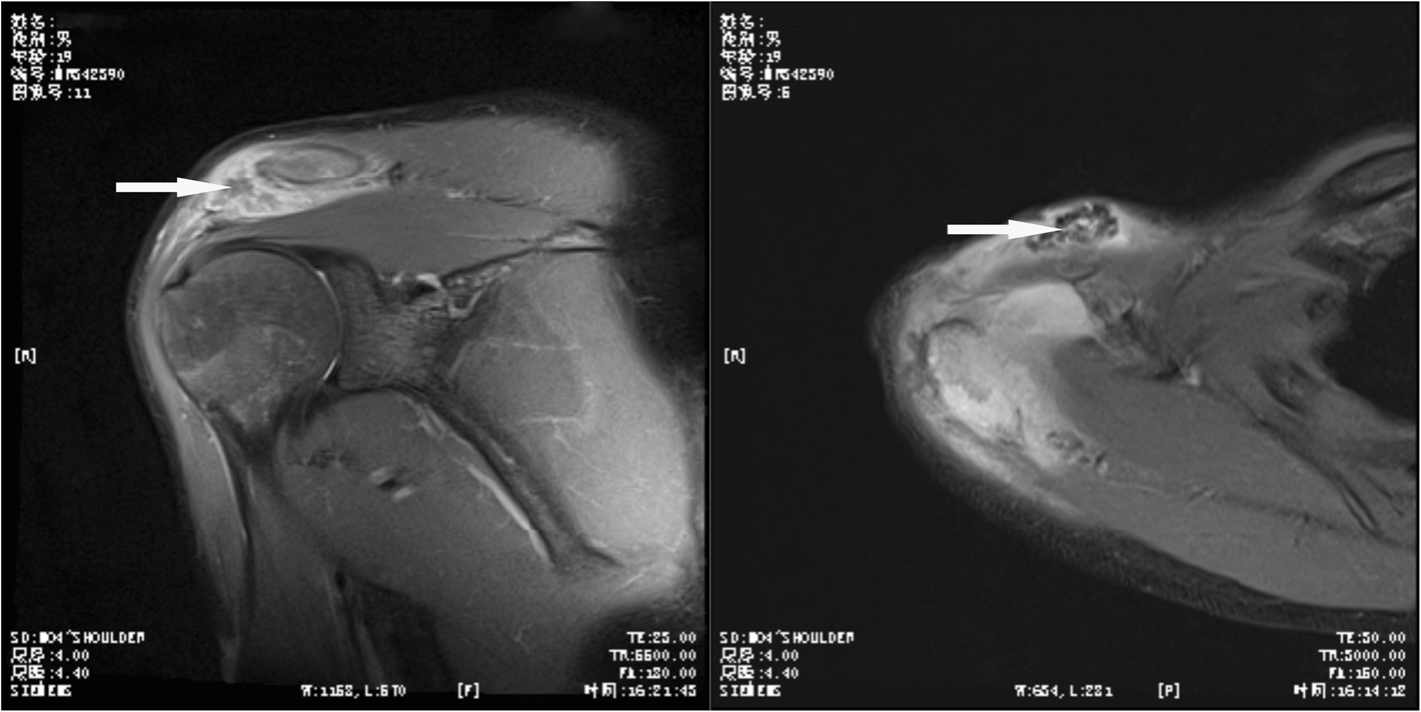
T2 weighted coronal and cross-sectional images showing involvement of acromioclavicular joint

On X-ray Radiograph and Computed Tomography (CT) of the right shoulder, obvious lytic destructions were observed at the acromion process of scapula without periosteal reaction (Figs. 1 and 2). Radiological examinations (X-ray and CT) of the chest revealed no abnormal finding. Magnetic Resonance Imaging (MRI) of the right shoulder showed marrow edema in the acromion appearing hyperintense on T2 weighted and spectral adiabatic inversion recovery (SPAIR) sequences, and ill-defined area of mixture of hyperintense and hypointense in the acromioclavicular joint suggested an inflammatory process. MRI of the area showed destruction of the acromioclavicular joint, and it was considered to be suppurative arthritis or tuberculous arthritis (Fig. 3).

Routine blood investigations were normal, except for a raised erythrocyte sedimentation rate (ESR) of 43 mm/h and C-reactive protein (CRP) of 39.1 mg/L. Human immunodeficiency virus (HIV) serological examination was negative. Canary yellow pus, found in the cavity, was sent for culture and Ziehl-Neelsen (ZN) stain to detect tubercle bacillus. However, no growth in culture and none acid fast bacilli (AFB) were seen. The sputum smear for AFB examined was negative, but detection of tuberculosis infection by T cell and purified protein derivative (PPD) skin test were positive, so presumptive therapy for tuberculosis disease was initiated: the patient was commenced on Rifampicin (RIF) and Isoniazid (INH) daily.

**Fig. 4:**
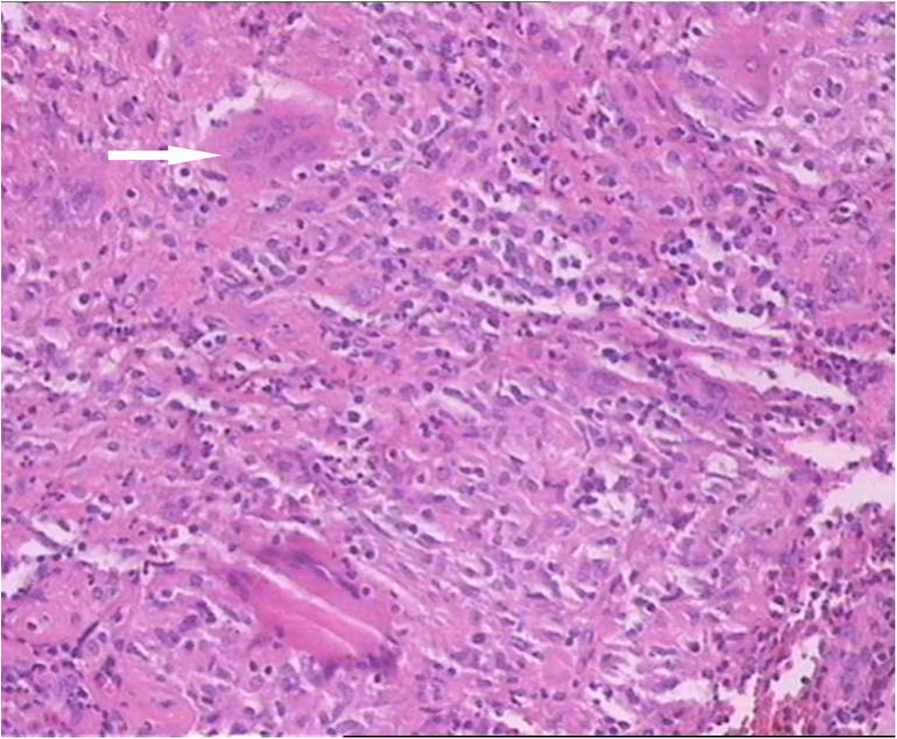
The pathological examination showing granulomatous inflammation with caseous necrosis(HE*100)

Debridement and vacuum sealing drainage (VSD) were applied on the right shoulder in one-stage operation. During the operation, a large amount of yellowish pus was found in the sinus, accompanied with caseous necrotic substances. The cavity in the sinus was through to the acromioclavicular joint, and part of the articular capsule was hyperemia and edema. Meanwhile, the acromion process of scapula was destroyed, and the bone debris could be found within the joint, but the lateral end of the clavicle remained intact. The bone debris was thoroughly debrided and the synovial membrane was excised during the operation. VSD was applied on the wound to continuous drainage after debridement, and the wound was irrigated with saline solution through the VSD tube after the operation. The necrotic tissues removed during the operation were sent to pathological examination, some specimens were sent for bacterial culture and ZN stain. The pathological examination showed granulomatous inflammation accompanied by caseous necrosis (Fig. 4), but the culture of specimens was negative. Multidrug anti-tubercular therapy was applied after the operation: Rifampicin (RIF), Isoniazid (INH), Pyrazinamide (PZA), Ethambutol (EMB). The granulation tissue in the wound was fresh with little exudate after removing the VSD 1 week later. Then the wound was sutured directly after debridement. Two weeks following surgery, the wound healed well and the patient gained full range of movement of the shoulder. Before discharge, ESR was descended at 15 mm/h and CRP at 4.39 mg/L, which were normal. The patient continued treatment with four anti-tubercular drugs (RIF, INH, PZA and EMB) after discharge. At final 1 year follow up, the patient showed painless movement and normal range of motion, without evidence of disease recurrence.

## Discussion

Osteoarticular tuberculosis occurs mostly in the weight-bearing and vulnerable area. The most common site is vertebral column, followed by knee and hip joint [1, 2], which account for more than 95% of all cases [3]. The incidence of osteoarticular tuberculosis in the upper extremities is low, and that of acromioclavicular joint is rare. The first case of acromioclavicular joint tuberculosis was reported in 1950 [4], in a series of 1074 unusual lesions of tuberculosis, there was just one case of acromial end of clavicle involvement. Since the first acromioclavicular joint tuberculosis was reported, there have been less than 10 relevant case reports.

In the past, osteoarticular tuberculosis was usually secondary to pulmonary tuberculosis [1], and primary tuberculosis lesions were often found. In absence of pulmonary lesions, osteoarticular tuberculosis may not come to mind as the first diagnosis [5]. However, the epidemic tendency of osteoarticular tuberculosis is changing according to clinical reports in recent years. Most patients did not accompany by pulmonary tuberculosis and systemic symptoms [2], resulting in more difficult diagnosis of osteoarticular tuberculosis. It was prone to misdiagnosis and missed diagnosis, especially unusual lesions of tuberculosis. According to the report [6], the most common manifestations were longstanding pain and functional loss in 83% unusual lesions of osteoarticular tuberculosis, which were often difficult to distinguish from osteoarthritis. In some cases, painless cold abscess can be the only clinical manifestation for a long period of time [2]. In a retrospective study [7], out of 16 patients of glenohumeral tubercular osteoarthritis 14 were misdiagnosed as frozen shoulder, and the mean prediagnostic time period to come to a correct diagnosis was 14.5 months. The delayed diagnosis resulted in poor prognosis of joint function.

The incidence of isolated acromioclavicular joint tuberculosis is extremely low. The disease is occult and has no typical tuberculosis symptoms, and it may present with symptoms of painless swelling of the joint or as a discharging sinus [8, 9]. Although the joint is swollen, the skin is mostly without redness and fever, which is different from common bacterial infection: this feature is the characteristic of tuberculous arthritis [2]. In this case, the lesion started as a papule with mild pain that subsequently ulcerated with purulent discharge, and antibiotic therapy was ineffective. Than we considered the possibility of tuberculosis, and completed imaging, cytology, pathology and other related examinations as soon as possible. Because of timely diagnosis and treatment, the patient avoided loss of joint function.

Imaging examination is an important method to diagnose osteoarticular tuberculosis, especially the application of MRI, which may help in the early diagnosis of tuberculosis and differentiation from osteomyelitis [10, 11]. Because MRI can show the severity of tuberculosis and has the characteristics of noninvasive and convenient, it plays an increasingly important role in the diagnosis of osteoarticular tuberculosis [12, 13]. In addition, it is essential to keep a high level of suspicion of tuberculosis at rare sites in order to make an early diagnosis [2, 14]. Tuberculosis should be considered as a differential diagnosis in all atypical presentations of osteoarthrosis, especially in China and India which remain as on the high burden country list for tuberculosis by WHO [15]. But Mittal R et al. [8] thought that although the presentation was as a lytic lesion with periarticular osteoporosis on radiography, the definitive diagnosis of osteoarticular tuberculosis was only made based on histological or bacteriological confirmation.

Debridement is a common method to treat osteoarticular tuberculosis, especially for early tuberculosis [16]. We believe that this operation is one of the most effective methods for the treatment of acromioclavicular joint tuberculosis, because this site is non-weight bearing and micro-motion joint, bone defect after debridement has little effect on function of the joint. Thorough debridement is the key to good prognosis. It is not recommended to suture directly if the wound is large and has much exudation, and VSD can be applied to continuous drainage after debridement. The drainage of secretions by VSD is more unobstructed than routine dressing change, which is beneficial to wound healing. In addition, it should be combined with standardized and continuous anti-tubercular chemotherapy to reduce the recurrence of tuberculosis.

## Conclusions

Osteoarticular tuberculosis is a great masquerader presenting in varied forms and in atypical locations. With the change of tuberculosis prevalence, osteoarticular tuberculosis can be a primary and isolated lesion, without active pulmonary tuberculosis or previous history of tuberculosis. Isolated acromioclavicular joint tuberculosis has been reported rarely and it is prone to misdiagnosis and missed diagnosis. For unexplained joint swelling and chronic shoulder pain, it is essential to keep tuberculosis in mind when conventional treatment is ineffective. This case demonstrates how clinical index of suspicion, appropriate and timely imaging examinations, and prompt surgical intervention result in optimum outcome. It can be said that in an endemic country, osteoarticular tuberculosis should be considered as a differential diagnosis in all atypical presentations in order to make early diagnosis which avoids residual problems.

## Abbreviations

AFB: Acid fast bacilli
CRP: C-reactive protein
CT: Computed Tomography
EMB: Ethambutol
ESR: Erythrocyte sedimentation rate
HIV: Human immunodeficiency virus
INH: Isoniazid
MRI: Magnetic Resonance Imaging
PPD: Purified protein derivative
PZA: Pyrazinamide
RIF: Rifampicin
SPAIR: Spectral adiabatic inversion recovery
VSD: Vacuum sealing drainage
ZN: Ziehl-Neelsen
